# Unpacking COVID-19 Vaccine Attitudes: Exploring Hesitancy and Acceptance Among Undergraduate Students in Bangladesh

**DOI:** 10.7759/cureus.49576

**Published:** 2023-11-28

**Authors:** Abhishek Bhadra, Vivek Podder, Md. Mynul Islam, Smriti Devnath, Imtiaz Hafiz, Kishwar Jahan Chowdhury, Hasnat Sujon, Md Rakibul Islam, Fahim Mohammed Ali, Thomas Ikechukwu Odo, Mahbuba Sudrul, Sabyasachi Roy, Anindita Dey, Farzana Hossain, Sanjay Kirshan Kumar, Abhishek Agarwala, Nadira Sultana Kakoly

**Affiliations:** 1 Pharmacology, Popular Medical College Hospital, Dhaka, BGD; 2 Medical Oncology, Miami Cancer Institute, Miami, USA; 3 Applied Statistics and Data Science, Institute of Statistical Research and Training, University of Dhaka, Dhaka, BGD; 4 Physiology, Popular Medical College Hospital, Dhaka, BGD; 5 Public Health, University of Eastern Finland, Kuopio, FIN; 6 Institute of Forestry and Environmental Sciences, University of Chittagong, Chittagong, BGD; 7 Infectious Disease, Directorate General of Health Services, Ministry of Health and Family Welfare, Dhaka, BGD; 8 Infectious Disease, Infectious Diseases and One Health Program, Hannover Medical School, Hannover, DEU; 9 Trauma and Orthopaedics, Tameside and Glossop Integrated Care NHS Foundation Trust, Ashton-under-Lyne, GBR; 10 Public Health, North South University, Dhaka, BGD; 11 Diabetes and Endocrinology, Royal Surrey County Hospital, Guildford, GBR; 12 Internal Medicine, Frimley Park Hospital, Frimley, GBR; 13 Gastroenterology, Bahria University of Health Sciences, Karachi, PAK; 14 Computer Science and Engineering, Bangladesh University of Engineering and Technology, Dhaka, BGD

**Keywords:** student education, vaccine hesitancy, covid-19 vaccine, vaccine science and policy, covid 19

## Abstract

Background: Vaccine hesitancy is a significant global health concern, and mass vaccination is essential in preventing the spread of COVID-19. Undergraduate students need to be prioritized for vaccination as they continue their academic curriculum physically. However, limited research explores vaccine hesitancy and acceptance among undergraduate students in Bangladesh. Therefore, this study evaluated vaccine hesitancy and acceptance among this population.

Method: A web-based cross-sectional study was conducted between May and June 2021 using a structured questionnaire to assess COVID-19 vaccine hesitancy and acceptance among undergraduate students in Bangladesh. The Oxford Covid-19 Vaccine Hesitancy Scale was used to measure vaccine hesitancy. The study used convenient sampling.

Result: Across the country, 334 undergraduate students participated in this study on COVID-19 vaccine acceptance, with a mean age of 22.4 years. Most participants were male and unmarried, most having spent four years at university. 89.52% of participants would accept a COVID-19 vaccine if it were suggested by educational institutions or available, while 4.49% refused to receive the COVID-19 vaccine. Participants showed low levels of vaccine hesitancy, with a mean score of 10.77 on the Oxford COVID-19 Vaccine Hesitancy Scale. Most participants had a positive attitude towards receiving the vaccine, with the majority wanting to get it as soon as it becomes available. No association was found between vaccine acceptance and participants' background characteristics.

Conclusion: Our study found a high level of vaccine acceptance among undergraduate students in Bangladesh, indicating that this group can be vaccinated quickly, significantly accelerating vaccination goals. However, further large-scale studies are recommended among vulnerable groups, including school and college students, to ensure vaccine preparedness.

## Introduction

The COVID-19 pandemic has forced countries worldwide to implement various non-pharmaceutical interventions (NPIs) to curb the virus's spread. Despite these efforts, the prevalence of COVID-19 cases continues to rise globally, and immunization remains the most effective way to prevent transmission [1-2]. However, developing SARS-CoV-2 vaccines in record time has created widespread apprehension, leading to vaccine hesitancy, which is currently hampering vaccination rollouts [3].

Since the emergence of SARS-CoV-2, several vaccines have been developed in an unprecedented 11-month timeframe [4-5]. However, the record-breaking speed of vaccine development has created widespread concerns, which currently impact the vaccine roll-out to combat the spread of SARS-CoV-2 [5]. Vaccine hesitancy, defined as the delay in accepting or refusing "vaccines despite the availability of vaccine services," remains a persistent issue [5-6]. Vaccine reluctance is a complex phenomenon that varies depending on factors such as time, place, and vaccine type [6]. These factors include contextual influences, such as historical, sociocultural, environmental, health system/institutional, economic, or political factors, as well as individual and group effects, such as personal perceptions of the vaccine (e.g., knowledge, awareness, conspiracy beliefs, attitudes, or personal experience with a vaccinated family member/friend), and vaccine influences (e.g., prices, mode of distribution, mode of management, strength, and information of healthcare employees, risks, or benefits).

Several countries have researched COVID-19 vaccine hesitancy. Indonesia and China reported the highest acceptance rates at 93% and 91%, respectively [7-9]. These Asian countries have a high level of trust in their governments, which may explain the high acceptance rates [10]. In the United States, vaccine acceptance rates among adults ranged from 57.5% to 68.5% [10], while medical and dental students reported acceptance rates of 75.5% [11] and 56% [12], respectively. The general public reported that acceptance rates were 78% [13]. Unfortunately, South Asian communities have some of the lowest acceptance rates worldwide, with acceptance rates of 53% in Pakistan [14], 65.8% in India, 63% in Afghanistan [15], and 74.6% in Bangladesh [16].

Students in universities and medical colleges play a vital role in society, as they are typically considered perceptive, influential, educated, and receptive to public health concerns [17]. Several studies have been conducted among medical and university students and the general population [18-21] to assess their willingness to receive the COVID-19 vaccine globally. However, there are limited studies on this topic in Bangladesh [22]. Despite the Bangladeshi government's efforts to vaccinate the general population, students were not yet eligible for vaccination due to their age at the time of the study. Therefore, this study aimed to assess the willingness and hesitancy of COVID-19 vaccination among undergraduate students in Bangladesh.

## Materials and methods

Study design

We conducted a cross-sectional study of undergraduate students in Bangladesh, utilizing a web-based survey administered between June and August 2021. We utilized a respondent-driven sampling technique to identify and recruit participants willing to complete the survey.

Questionnaire development

To assess the COVID-19 vaccine hesitancy and acceptance among undergraduate students in Bangladesh, we utilized a structured questionnaire divided into four sections: socio-demographic information, academic profile, hesitancy scale, and acceptance of the COVID-19 vaccine. The Oxford COVID-19 Vaccine Hesitancy Scale [19], a validated seven-item measure used to assess vaccine hesitancy among adults in the UK, was employed in this study. The scale's scores range from 7 to 35, with higher scores indicating greater vaccine hesitancy. In addition to employing the Oxford COVID-19 Vaccine Hesitancy Scale [19], our study incorporated a direct measure of vaccine acceptance. We used the following question from the questionnaire of a previous study-"Would you accept the COVID-19 vaccine if generally available, as recommended by your institution?" The question allowed participants to express their level of agreement with the statement, offering a range of responses from 'Completely agree' to 'Completely disagree,' along with an option for 'Don't know.' Such a Likert scale is widely utilized in survey research to quantify attitudes and opinions [20]. 

The survey aimed to include a diverse student population by employing a bilingual questionnaire in both English and Bengali. Recognizing English as the primary language in medical schools and widely used across various disciplines, this approach ensured accessibility to a broad range of students. A meticulous comparison of both language versions was conducted, addressing discrepancies through collaborative discussions within the research team to enhance clarity and cultural appropriateness.

Inclusion and exclusion criteria

The study exclusively enrolled unvaccinated undergraduate students from diverse regions and institutions across Bangladesh, focusing on ensuring a diverse representation of age and sex. Inclusion criteria comprised individuals within the undergraduate level who had not received the COVID-19 vaccine. The study excluded postgraduate students and those below the 12th standard in Bangladesh. Additionally, individuals who had already been vaccinated against COVID-19 were not considered eligible for participation.

Study procedure

To optimize our sample size, we widely distributed our web-based structured questionnaire. Utilizing Google Forms, the questionnaire was electronically disseminated to undergraduate and medical students through dedicated Facebook groups, specifically tailored to students from distinct academic years within their respective institutions. To ensure data integrity, we generated a unique Google Form link, enabling participants to complete the survey online while implementing measures to restrict one response per email and prevent multiple entries from the same individual. Additionally, participants were required to answer pertinent questions for inclusion in the analysis.

**Figure 1:**
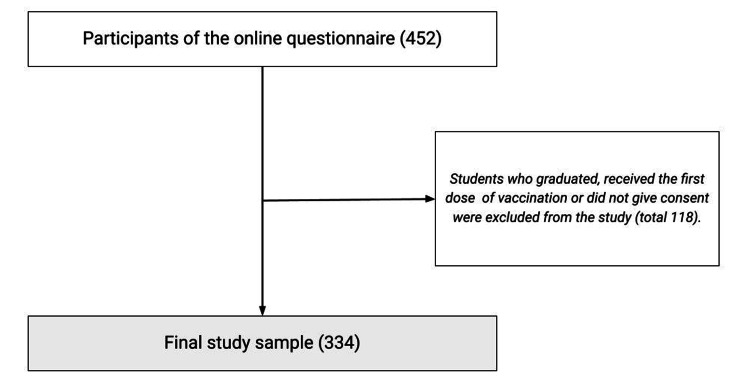
Flow chart showing the sampling technique

Participation in our study was voluntary and anonymous, with participants providing only their demographic information. Participating students were encouraged to share the survey within their peer networks at the same institutions to enhance reach. Notably, no monetary or other incentives were offered for survey completion. A total of 452 students responded initially, yet following the exclusion of incomplete responses, our final dataset comprised 334 samples for analysis (Figure 1).

Statistical analysis

The analysis focused on the distribution of categorical variables derived from the questionnaire responses. To this end, the study employed frequency distribution methods to present how responses were distributed across different categories for each question. The association between different categorical variables was determined using the Pearson chi-square test. Hesitancy was measured using the Oxford COVID-19 Vaccine Hesitancy Scale [19]. Mean scores were calculated by adding the responses to each scale question, coded from 1 to 5. We excluded the "Do not know" option from scoring question-specific responses. A higher score indicates a higher level of hesitancy to take a vaccine. All data analyses were performed using STATA 16.0 and IBM SPSS Statistics 25, which were chosen for their robust capabilities in handling categorical data analysis.

Ethical approval and consent

The study has obtained approval from the Ethical Review Committee (ERC) at North South University, Bangladesh, with the reference number 2021/OR-NSU/IRB/0501. Consent was obtained by all participants in this study.

## Results

**Figure 2:**
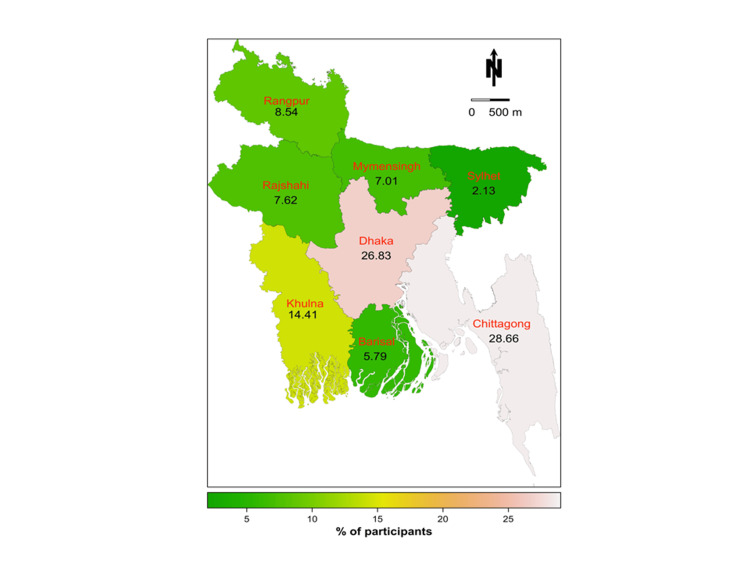
Demographic distribution (division-wise distribution) of the study participants in Bangladesh

Three hundred thirty-four students, with a mean age of 22.38, completed the survey. Male participants (57.78%) outnumbered females, and unmarried respondents (95.21%) outnumbered married respondents. Throughout the entire nation, the Chattogram division had the largest percentage of students participating (28.66%), while Sylhet had the lowest percentage (2.13%) (Figure 2). Regarding academic profiles, most participants (107) had spent four years at university. Of the participants, 25.15% had medical backgrounds, 14.07% had backgrounds in engineering, and the remaining students (60.78%) were enrolled in universities (except engineering students).

**Figure 3:**
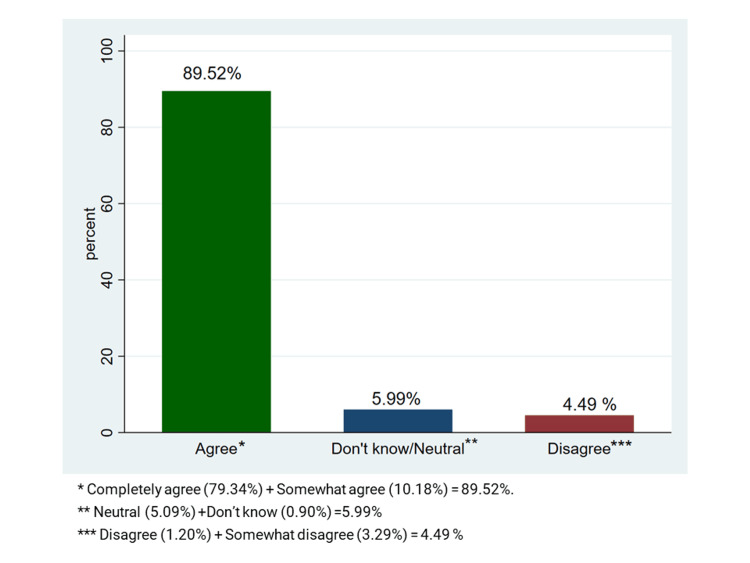
The acceptance of COVID-19 vaccine among the participants

A COVID-19 vaccination would be accepted by 89.52% of participants if educational institutions suggested it or if it was available to get; of these, 79.34% were fully in agreement, and 10.18% agreed with having some confusion. Contrarily, 4.49% of participants refused to receive the vaccination, while 5.99% were unable to decide on receiving the COVID-19 vaccine (Figure 3).

Besides higher vaccine acceptance among the students, the study depicted lower hesitancy to receive the COVID-19 vaccine among them by the lower mean of 10.77 (± 4.71) on the Oxford COVID-19 Vaccine Hesitancy Scale. A higher score indicates a higher level of vaccine hesitancy, ranging from 7 to 35 (Table 1). To describe the response of the scale in-depth, most participants (71.86%) were willing to take a COVID-19 vaccine if offered, with 202 out of 334 wanting to get the vaccine as soon as possible. Most participants (83.83%) had a positive attitude towards receiving a vaccine, with 195 participants wanting to get it as soon as it becomes available at the local pharmacy. Additionally, 88.63% of students are likely to encourage their family or friends to take the COVID-19 vaccination if they want to. More than half of the students (56.89%) were eager to get a COVID-19 vaccine, and 62.28% think taking a COVID-19 immunization is essential. Therefore, most participants responded to the seven questions of the Oxford Vaccine Hesitancy Scale with less hesitancy (Table 2). Vaccine acceptance did not demonstrate any statistically significant correlation with age, gender, marital status, or years of academic study (Table 1). 

**Table 1:** Socio-demographic, academic and COVID-19 exposure-related Characteristics of the participants N: number; SD: standard deviation

Characteristics		Frequency (%) / Mean ± SD	Vaccine Acceptance			P-value (Chi-square)
			Agree/ somewhat agree n (%)	Do not know/ neutral n (%)	Disagree n (%)	
Age (year)		22.38 ± 1.97				
Gender						
Male		193 (57.78)	172 (89.10%)	14 (7.30%)	7 (3.60%)	0.369
Female		141 (42.22)	127 (90.10%)	6 (4.30%)	8 (5.70%)	
Marital Status						
Married		16 (4.79)	15 (93.80%)	0 (0.00%)	1 (6.30%)	0.561
Unmarried		318 (95.21)	284 (89.30%)	20 (6.30%)	14 (4.40%)	
Year(s) of studying						
< 1 year		36 (10.91)	35 (97.20%)	1 (2.80%)	0 (0.00%)	0.266
1 year		39 (11.82)	34 (87.20%)	2 (5.10%)	3 (7.7%)	
2 years		46 (13.94)	41 (89.10%)	2 (4.30%)	3 (6.50%)	
3 years		71 (21.52)	66 (93.00%)	3 (4.20%)	2 (2.80%)	
4 years		107 (32.42)	96 (86.50%)	11 (9.90%)	4 (3.60%)	
5 years		20 (6.06)	16 (80.00%)	1 (5.00%)	3 (15.00%)	
> 5 years		11 (3.33)	11 (100.00%)	0 (0.00%)	0 (0.00%)	
Faculty						
Engineering		47 (14.07)	44 (93.60%)	1 (2.10%)	2 (4.3%)	0.27
Medical		84 (25.15)	72 (85.70%)	9 (10.70%)	3 (3.60%)	
Other		203 (60.78)	183 (90.10%)	10 (4.90%)	10 (4.90%)	

**Table 2:** Oxford vaccine hesitancy scale N: number; SD: standard deviation; COVID-19: Coronavirus Disease 2019

Item	Response	Frequency (n)	Percentage
1. Would you take a COVID-19 vaccine (approved for use in Bangladesh) if offered?	Definitely	240	71.86
Probably	48	14.37	
I may or I may not	31	9.28	
Probably not	7	2.1	
Definitely not	5	1.5	
Don't know	3	0.9	
2. If there is a COVID-19 vaccine available:	I will want to get it as soon as possible	202	60.48
I will take it when offered	85	25.45	
I'm not sure what I will do	30	8.98	
I will put off (delay) getting it	5	1.5	
I will refuse to get it	5	1.5	
Don't know	7	2.1	
3. I would describe my attitude towards receiving a COVID-19 vaccine as:	Very keen	154	46.11
Pretty positive	126	37.72	
Neutral	24	7.19	
Quite uneasy	22	6.59	
Against it	6	1.8	
Don't know	2	0.6	
4. If a COVID-19 vaccine was available at my local pharmacy, I would:	Get it as soon as possible	195	58.38
Get it when I have time	56	16.77	
Delay getting it	17	5.09	
Avoid getting it for as long as possible	24	7.19	
Never get it	20	5.99	
Don't know	22	6.59	
5. If my family or friends were thinking of getting a COVID-19 vaccination, I would:	Strongly encourage them	175	52.4
Encourage them	121	36.23	
Not say anything to them about it	14	4.19	
Ask them to delay getting the vaccination	10	2.99	
Suggest that they do not get the vaccination	4	1.2	
Don't know	10	2.99	
6. I would describe myself as:	Eager to get a COVID-19 vaccine	190	56.89
Willing to get the COVID-19 vaccine	95	28.44	
Not bothered about getting the COVID- 19 vaccine	36	10.78	
Unwilling to get the COVID-19 vaccine	7	2.1	
Anti-vaccination for COVID-19	2	0.6	
Don't know	4	1.2	
7. Taking a COVID-19 vaccination is (Mean ± SD)	10.77 (± 4.71)		

## Discussion

Our study provides valuable insights into the acceptance of the COVID-19 vaccine among Bangladeshi undergraduate students. Our findings suggest a high level of vaccine acceptance, with 89.5% of students indicating their willingness to accept the vaccine. This result is consistent with previous studies conducted in Asian nations, indicating vaccine acceptance rates exceeding 80%. However, there were slight variations in acceptance rates among different countries. This rate differs slightly from that of neighboring countries like Pakistan (53%) [14], India (65.8%) [18], and Afghanistan (63%) [15], but is comparable to the rates observed in China (91%) and Indonesia (93%) [8-9].

Our findings are consistent with those of Haque et al. [21], which reported a positive correlation between higher educational levels and vaccine acceptance. Our findings revealed a significant rise in vaccine acceptance among our undergraduate study population. This increase was from 74.6% [16] to 89.5% during Bangladesh's second wave of COVID-19. It is crucial to note that our study's cohort is comprised entirely of undergraduate students, a demographic still in the process of completing their higher education. By focusing on this specific educational demographic, our study contributes to understanding vaccine acceptance among a crucial population segment at a formative stage of their academic and professional careers. This demographic's high vaccine acceptance rate is an encouraging sign and underscores the potential impact of educational institutions in driving vaccination efforts. 

The higher acceptance rate of the COVID-19 vaccine among Bangladeshi undergraduate students during the second wave may be attributed to their direct observation of peers and family members receiving the vaccine without adverse effects. This observation likely influenced their perception of the vaccine's safety and efficacy. Additionally, understanding the perspectives of undergraduate students on vaccine acceptance is crucial, as they represent one of the priority groups for vaccination during the pandemic.

Limitation

Despite its contributions, our study has several limitations that warrant consideration. First, our focus solely on undergraduate students may restrict the generalizability of our findings to other demographic groups, such as high school or postgraduate students. Consequently, our sample may not fully represent the broader population's attitudes toward vaccination. Additionally, our reliance on online data collection could introduce a selection bias, as it inherently excludes individuals without internet access. This method may not accurately capture the full spectrum of opinions regarding vaccination, particularly among those who lack digital connectivity.

Furthermore, the convenience sampling approach and online survey method may further contribute to biases in data collection. Such biases could skew the representation of attitudes and behaviors in our study population. Lastly, our study's scope did not extend to exploring the underlying causes of vaccine hesitancy, which limits a deeper understanding of the specific factors influencing undergraduate students' attitudes toward vaccination, which could be crucial in addressing hesitancy effectively.

Strength

Despite these limitations, our study offers notable strengths. We achieved a representative sample of Bangladeshi undergraduate students covering diverse regions and major faculties. The observed high vaccine acceptance rate of 89.5% among the participants indicates a positive inclination towards vaccination within this demographic. This finding is particularly significant given the critical role of this population in vaccination strategies. Additionally, by placing our findings in the context of similar studies conducted in other Asian countries, we provide a broader perspective on vaccine acceptance rates in the region. This comparative approach enriches understanding of regional differences and similarities in vaccine acceptance. Using a validated measure, the Oxford COVID-19 Vaccine Hesitancy Scale enhances the reliability of our measurements.

Furthermore, our insights into undergraduate students' attitudes, a priority group for vaccination, contribute valuable information for public health interventions. The insights gleaned can inform targeted, efficient, and potentially more cost-effective vaccination strategies, such as campaign-based or immediate vaccination processes specifically tailored to this group. This practical application underscores the relevance and utility of our research in informing public health policies and vaccination campaigns.

Recommendation

With a high acceptance rate among undergraduate students, tailored policies and interventions can harness this positive trend for effective COVID-19 vaccination strategies. Implement targeted vaccination campaigns through university channels, establish on-campus clinics for accessibility, and organize educational workshops to dispel myths and reinforce the importance of vaccination. Utilize peer influence programs, incorporate positive experiences from vaccinated students, and integrate vaccination discussions into academic culture. Develop crisis communication plans to address concerns transparently and foster trust. Monitor acceptance rates, collaborate with student organizations, and engage healthcare providers for seamless vaccination processes. Consider incentive programs to motivate students further. Recommendations include ongoing research to understand evolving attitudes and leveraging partnerships with local businesses for additional incentives. A comprehensive, student-focused approach can maximize vaccine uptake and contribute to a safer campus environment.

## Conclusions

The study findings indicate that most undergraduate students from medical, engineering, and university backgrounds had a positive attitude toward accepting the COVID-19 vaccination. Despite some limitations, this study provides valuable insights into vaccine hesitancy among this demographic in Bangladesh. Based on the high acceptance rate observed in this study, a campaign-based or immediate vaccination process targeting undergraduate students could be a cost-effective and time-saving approach. However, further research is recommended to be conducted to target other specific populations, such as high school and college students. It is essential for government officials to continue implementing multidisciplinary schooling, pro-vaccination awareness initiatives, and health seminars to maintain a positive attitude toward vaccine uptake.
